# Chaos control in cardiac dynamics: terminating chaotic states with local minima pacing

**DOI:** 10.3389/fnetp.2024.1401661

**Published:** 2024-07-03

**Authors:** Daniel Suth, Stefan Luther, Thomas Lilienkamp

**Affiliations:** ^1^ Computational Physics for Life Science, Nuremberg Institute of Technology Georg Simon Ohm, Nuremberg, Germany; ^2^ Max Planck Institute for Dynamics and Self-Organization, Göttingen, Germany; ^3^ German Center for Cardiovascular Research (DZHK), Partner Site Göttingen, Göttingen, Germany; ^4^ Institute for the Dynamics of Complex Systems, Georg-August-Universität Göttingen, Göttingen, Germany; ^5^ Institute of Pharmacology and Toxicology, University Medical Center Göttingen, Göttingen, Germany

**Keywords:** defibrillation, arrhythmias, chaos control, excitable media, numerical simulation, feedback-controlled protocol, network physiology, cardiovascular network

## Abstract

Current treatments of cardiac arrhythmias like ventricular fibrillation involve the application of a high-energy electric shock, that induces significant electrical currents in the myocardium and therefore involves severe side effects like possible tissue damage and post-traumatic stress. Using numerical simulations on four different models of 2D excitable media, this study demonstrates that low energy pulses applied shortly after local minima in the mean value of the transmembrane potential provide high success rates. We evaluate the performance of this approach for ten initial conditions of each model, ten spatially different stimuli, and different shock amplitudes. The investigated models of 2D excitable media cover a broad range of dominant frequencies and number of phase singularities, which demonstrates, that our findings are not limited to a specific kind of model or parameterization of it. Thus, we propose a method that incorporates the dynamics of the underlying system, even during pacing, and solely relies on a scalar observable, which is easily measurable in numerical simulations.

## 1 Introduction

Sudden cardiac death is the cause of 15%–20% of all deaths in western societies, making it a major health concern (Wong et al., 2019). Among the other types of cardiac arrhythmias, atrial fibrillation does not usually pose an immediate threat to life, but it is the most common form of sustained cardiac arrhythmia and often leads to strokes (Wolf et al., 1978). In contrast, ventricular fibrillation proves to be lethal if it is not stopped within minutes and is the most common cause of death in industrialised countries (Myerburg et al., 1997). Cardiac arrhythmias in network physiology are caused by chaotic patterns of the electrical excitation wave dynamics in the myocardium, that are not yet fully understood (Gray et al., 1998). Terminating cardiac arrhythmias like atrial and ventricular fibrillation is an ambitious task and most ofen requires the application of a high-energy electric shock (Ammannaya, 2020; Cheskes et al., 2022). As considerable electrical currents are induced in the myocardium, this approach involves severe side effects like post-traumatic stress (Godemann et al., 2004; Sears et al., 2011) and an increasing severity of life-threatening myocardial disorders after resuscitation (Xie et al., 1997; Tang et al., 2000).

We refer to defibrillation/pacing as the application of controlled electrical stimuli through an external electric field to counter spiral/scroll wave dynamics in the electrical excitation of the myocardium (Davidenko et al., 1995) with the ultimate goal to terminate all chaotic patterns in the heart rhythm. After successful termination of all chaotic patterns of the transmembrane potential of the heart, the sinus rhythm can be reinitiated by the electrical system of the heart.

There are numerous protocols for the control of chaotic patterns in excitable media with the aim of reducing the energy required for successful defibrillation. It is possible to apply stimuli at certain locations in the heart to terminate all chaotic patterns. For example, DeTal et al., 2022 identified specific isolines of the refractory backs of spiral waves as vulnerable locations. If stimuli are applied solely at these locations, the underlying dynamics is terminated. Lilienkamp and Parlitz, 2020 demonstrated that, in principle, it is sufficient to apply stimuli at isolated spots for successful termination. Otani et al., 2019 performed 3D simulations on a simplified ventricular geometry, to show the impact of the direction of an external electrical field on the success rate for defibrillation. Other protocols aim to apply either single stimuli at specific points in time (Trayanova, 2006; Steyer et al., 2023) or pulse sequences at predefined periods/frequencies (Fenton et al., 2009; Janardhan et al., 2014; Buran et al., 2017; Hornung et al., 2017; Lilienkamp et al., 2022a; Lilienkamp et al., 2022b; Aron et al., 2023). The timing of pulses can also be determined by feedback-controlled protocols, which incorporate the underlying dynamics and the reaction of the system to stimuli. Possible observables suitable for this group of algorithms were discussed by Buran et al., 2023 whereby the length of the refractory boundary has already been tested as a possible observable for a feedback-controlled pacing strategy (Buran, 2023). Numerical investigations of all these approaches promise reductions of the energy required for successful defibrillation and thus mitigate the aforementioned negative side effects of a conventional defibrillation. However, it remains unclear whether further energy reductions are in principle possible.

Numerical simulations of defibrillation attempts vary drastically in the level of detail of the underlying model. Striving to achieve a patient-specific level of parameterization, numerical simulations can be tailored accordingly, which involves the usage of the patients real heart geometry (Trayanova and Rantner, 2014; Lee et al., 2018), the orientation of its muscle fibres (Helm et al., 2005; Bayer et al., 2012), the cardiac cell model (ten Tusscher et al., 2004; Bueno-Orovio et al., 2008), etc. This type of modelling aims to predict patient-specific results (Niederer et al., 2019; Gerach et al., 2021; Gillette et al., 2021). However, multiple groups develop and test new pacing protocols on simplified models to express the temporal evolution of the transmembrane potential in 2D without the incorporation of the fibre orientation (Buran et al., 2017; Lilienkamp and Parlitz, 2020; DeTal et al., 2022). The low computation effort of running these simplified simulations allows for a flexible exploration of novel methods across a broad range of parameterizations, encompassing diverse models and parameterizations of them. Basic mechanisms for successful defibrillation can be identified but the accuracy of the predictions (e.g., the exact energy reduction) with respect to *in vivo* or *ex vivo* experiments is limited.

In this study, we investigate a novel feedback-based approach that incorporates the dynamics of the heart during pacing, to further minimize the energy required for successful defibrillation and thus mitigate the negative side effects associated with high-energy defibrillation. Compared to the aforementioned pacing protocols, the pulse timings derived with this approach are therefore not predetermined but calculated during the pacing process, based on the response of the system to the previous stimuli. The proposed approach solely relies on the measurement of the mean value of the transmembrane potential, an observable that can be measured very easily in numerical simulations.

In Section 2, we introduce the modelling and numerical treatment of our control attempts as well as a novel pacing protocol. A statistical analysis of the amplitude reduction achievable with our control scheme based on multiple cardiac tissue models, a comparison of its working mechanisms with pre-existing approaches, and a sensitivity analysis are given in Section 3.

## 2 Materials and methods

In this section we present the modelling and numerical treatment of our control attempts as well as a novel pacing protocol.

### 2.1 Simulation of cardiac dynamics

This section provides the mathematical framework to describe the myocardium, which consists of a network of electrically and mechanically coupled cardiac cells.

#### 2.1.1 Basic equations and numerical setup

The underlying mono-domain equations to model the temporal evolution of the transmembrane potential *V*_m_ are given by (Tuckwell, 1988)

$$\partial_{t}V_{m}=\nabla \cdot \left(D\nabla V_{\text{m}}\right)-\frac{I_{\text{ion}}\left(V_{\text{m}},h\right)}{C_{\text{m}}},$$ (1)

$$\partial_{t}h=g\left(V_{\text{m}},h\right),$$ (2)

where *D* describes the diffusion tensor, *I*_ion_ the ionic currents through the cell membrane, *C*_m_ the membrane capacitance per unit membrane area, and $h={\left(h_{1},\ldots ,h_{\text{k}}\right)}^{T}$ the gating variables. The exact design of *I*_ion_, *g*, and *h* depends on the model of cardiac dynamics in use, as described in Section 2.1.2 and the Supplementary Material. For all simulations we used no-flux boundary conditions

$$\nabla V_{\text{m}}\cdot n=0,$$ (3)

(where *n* describes the unit vector normal to the boundaries) and assumed isotropic as well as homogeneous diffusion $\left(D\in R^{+}\right)$ . We solved the equation system described by (1)–(3) numerically using a GPU-based implementation on a two-dimensional grid using forward time-centered space (FTCS) method: A rectangular and equidistant spatial grid with a spacing of Δ*x* and *N* × *N* nodes using the finite difference method and fully explicit Euler method with Δ*t* (Press, 2007). To increase the numerical stability of the numerical scheme, we applied the Rush-Larsen (Rush and Larsen, 1978) method for the temporal integration of the gating variables. The chosen parameters Δ*x*, *N* × *N*, Δ*t*, and *D* meet the stability criterion of the FTCS scheme (Press (2007)) and were specified individually for each cardiac cell model to meet characteristic features (e.g., number of phase singularities) of the resulting chaotic states and can be found in Table 1.

**TABLE 1:** Modelling parameters for each cardiac cell model. Chaotic states can be characterized by the dominant frequency *f*_dom_ and the mean number of phase singularities *N*_PS_. Electrical stimuli of length *t*_pulse_ are modelled via the concept of AVEs with a size *S*_ave_ of a single AVE, where a number *N*_ave_ of AVEs cover a relative fraction *A*_cov_ of the domain. Δ*t*_median_ is the median time between a local minimum and the consecutive local maximum in the mean value of the transmembrane potential.

	AP	BOCF	FK	MSA
*N*	200	576	200	150
Δ*x* [mm]	0.8	0.8	1.0	0.8
Δ*t* [ms]	0.1	0.1	0.1	0.1
*D* [cm^2^/s]	1.5	2.0	2.0	0.5
*C*_m_ [mF/cm^2^]	1.0	1.0	1.0	1.0
*f*_dom_ [Hz]	22.46 ± 1.24	2.65 ± 0.04	6.28 ± 0.44	3.37 ± 0.03
*N*_PS_	22.11 ± 2.85	12.76 ± 5.37	10.74 ± 2.23	14.85 ± 3.98
*S*_ave_ [mm^2^]	0.5	0.8	2.0	0.8
*N*_ave_	12000	66356	2500	6750
*A*_cov_ [%]	30	20	25	30
*t*_pulse_ [ms]	2	2	2	2
Δ*t*_median_ [ms]	24	110	90	168

#### 2.1.2 Investigated models of cardiac dynamics

**FIGURE 1:**
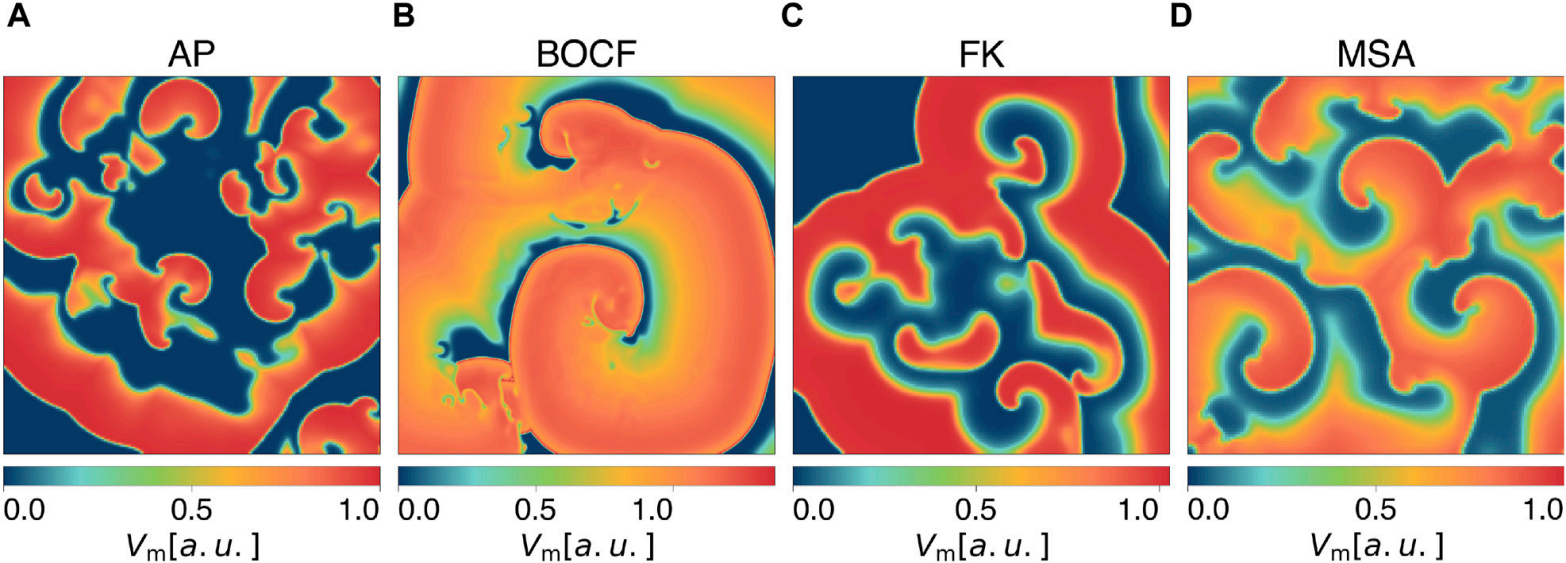
Snapshots of the membrane potential *V*_m_ during spiral wave chaos of **(A)** the Aliev-Panfilov (AP) model, **(B)** the Bueno-Orovio-Cherry-Fenton (BOCF) model, **(C)** the Fenton-Karma (FK) model, and **(D)** the Mitchell-Schaeffer model with the adaptions made by Alvarez et al. (MSA).

Models of cardiac dynamics describe the electrical action potential dynamics of cardiomyocytes (Clayton et al., 2011). Different models of cardiac dynamics can be used to model the electrical activity in the myocardium of different species or different types of arrhythmia and thus exhibit different properties in the underlying dynamics, such as the shape and duration of action potentials (Fenton and Karma, 1998; Bueno-Orovio et al., 2008; Mahajan et al., 2008). We carried out simulations with four different models: the Aliev-Panfilov model (AP) (Aliev and Panfilov, 1996), the Bueno-Orovio-Cherry-Fenton model (BOCF) (Bueno-Orovio et al., 2008), the Fenton-Karma model (FK) (Fenton and Karma, 1998), and the Mitchell-Schaeffer model (Mitchell and Schaeffer, 2003) with the adaptions made by Alvarez et al. (MSA) (Álvarez et al., 2012). We specifically included the latter model because it represents an exception as it models ischaemic cardiac tissue. Although the other models can reconstruct distinctive features of diverse arrhythmia types, their parameterization is primarily designed to align with the shape and duration of the action potential during a state of sinus rhythm. The models we used for our experiments differ from more complex ionic models (ten Tusscher et al., 2004; Luo and Rudy, 1994) by not modelling the behaviour of all ion channel currents individually, but by combining the transmembrane currents/ion concentrations in specified functions/gating variables. Our choice of models and their parameterizations (given in the Supplementary Material) result in a broad range of characteristic features of the underlying dynamics, such as the number of phase singularities and dominant frequencies (see Table 1). This is intended to show that our findings are not tailored to a specific model of cardiac dynamics, a parameterization of it, a single biological species, or one specific type of cardiac arrythmia but rather show a basic mechanism inherent in all of these models. Due to a lower computational effort, the usage of phenomenological models allows us to perform a large number of simulations and thus a statistical analysis of the presented methods on multiple models of cardiac dynamics. Exemplary snapshots of the transmembrane potential *V*_m_ during spiral wave chaos are shown in Figure 1 for each model, respectively. As done in previous publications on pacing protocols, we also neglect the influence of the fibre orientation in the myocardium, assume absence of scar tissue and except for the MSA model, healthy electrophysiological behaviour of the ion channels within the myocardium (Trayanova, 2006; Janardhan et al., 2014; Buran et al., 2017; Hornung et al., 2017; Lilienkamp and Parlitz, 2020; Lilienkamp et al., 2022a; Lilienkamp et al., 2022b; DeTal et al., 2022; Steyer et al., 2023).

#### 2.1.3 Initialization of spiral wave chaos

For each model of cardiac dynamics, ten initial conditions were created by applying spatially randomized stimulations to the transmembrane potential of a state already exhibiting a spiral wave pattern. After these stimulations, each state was evolved for at least 5 s to generate independent initial conditions. Each initial condition was tested for another 5 s, to exclude self-terminating states (Qu, 2006; Lilienkamp et al., 2017; Aron et al., 2019). This way, we ensure that successful termination attempts can be attributed to the corresponding pacing protocol. Characteristics of the underlying dynamics created this way are given in Table 1. Further details about the initialization of chaotic states and video material of it are given in the Supplementary Material.

#### 2.1.4 Termination attempts

We simplify the application of electrical stimuli by using the concept of artificial virtual electrodes: Heterogeneities in the electrical conductivity of the myocardium are responsible for the depolarization of the transmembrane potential induced by an external electric field (Efimov et al., 2000; Pumir et al., 2007; Luther et al., 2011; Bittihn et al., 2012). This underlying mechanism is modelled by the application of local current injections at predefined locations within the simulation domain (Lilienkamp et al., 2022b). These local current injections act as a network of local stimulation sites and are referred to as artificial virtual electrodes (AVEs). For each initial condition and each termination attempt, a fixed number *N*_ave_ of non-overlapping locations, of size *S*_ave_ are selected such that a relative fraction *A*_cov_ of the simulation domain is covered by local current injections. In previous publications, the coverage area *A*_cov_ was chosen from a broad range from 2.5% to 5% (Buran et al., 2017) to 25%–30% (Lilienkamp et al., 2022b) to 100% (Gray, 2002). The choice of *A*_cov_ is determined not only by the spatial resolution of the simulation domain but also by the decision which heterogeneities in the myocardium are modelled as virtual electrodes. We follow the abstraction of Lilienkamp et al., 2022b that different types of heterogeneities may contribute to the entirety of AVEs: blood vessels (Buran et al., 2017), lateral intercellular couping as well as extracellular resistive heterogeneities (Caldwell et al., 2015), boundaries of high curvature (Bittihn et al., 2012), and others.

The number of AVEs *N*_ave_, the size of each AVE *S*_ave_, and the resulting spatial fraction *A*_cov_ for each model of cardiac dynamics is given in Table 1. However, results for differenet values of *A*_cov_ are also presented in chapter 3.4, to emphasize the stability of our conclusion with respect to the exact choice of *A*_cov_. An exemplary excitation using an AVE mask is shown in Figure 3A. The locally injected current of each AVE was selected randomly from the interval [0, *A*_max_] to account for variations of depolarization intensity in living tissue due to the shape of heterogeneities. Relatively small heterogeneities, for example, are stimulated only at large external field strengths (Bittihn et al., 2012). By employing the concept of artificial virtual electrodes, we accept that in particular the first milliseconds of the interaction between an external electric field and the myocardium is simplified. However, this concept enables a grid spacing (Δ*x*, *N*, and Δ*t*) to perform a large number of simulations and thus a statistical analysis of the presented methods on multiple models of cardiac dynamics. The described methodology matches the one used by Lilienkamp et al., 2022b. To determine the success rate of a pacing protocol at a given amplitude *A*_max_, we performed ten termination attempts (each with different spatial distributions of AVEs) for each of the ten initial conditions. The success rate describes the fraction of successful attempts. A termination attempt is considered successful if after a period of 5˜*f*_dom_, the maximum of the transmembrane potential is below a threshold *V*_m,thresh_ = 0.01 [*a*.*u*.]. The dominant frequency *f*_dom_ is defined as the frequency with the largest amplitude in the power spectral density of the underlying dynamics.

### 2.2 Local minima pacing

Apart from defibrillation attempts with a single pulse (SinglePulse), numerous low energy pacing protocols exist that are based on the application of pulse sequences derived from variables of the frequency spectrum of the underlying dynamics. For example, pulses can be applied at a constant frequency (EquiDist) (Fenton et al., 2009; Luther et al., 2011; Janardhan et al., 2014; Lilienkamp et al., 2022a) or at decelerating pulse frequencies (ADP) (Lilienkamp et al., 2022b). Both protocols share the characteristic that all pulse frequencies (and thus pulse timings) are calculated based on observations of the system in the last seconds prior to the first pulse. During the pacing process, the dynamics of the system is neglected, as the predetermined pulse frequencies are kept fix. However, Steyer et al. showed that the exact timing of a single pulse plays a major role for a successful termination of a chaotic state (Steyer et al., 2023). Additionally, the accuracy of the calculated pulse frequencies is inherently limited by the time period for which the system is observed prior to pacing (B Traykov et al., 2012), as ADP and EquiDist are based on the frequency spectrum of the underlying dynamics and, respectively, its dominant frequency. Furthermore, Buran et al., 2023 observed that successful low energy defibrillation protocols are characterized by a coordinated interplay between the applied pulses and other observables of the underlying dynamics, such as the refractory boundary length, and proposed a feedback-controlled strategy based on this observable (Buran, 2023).

**FIGURE 2:**
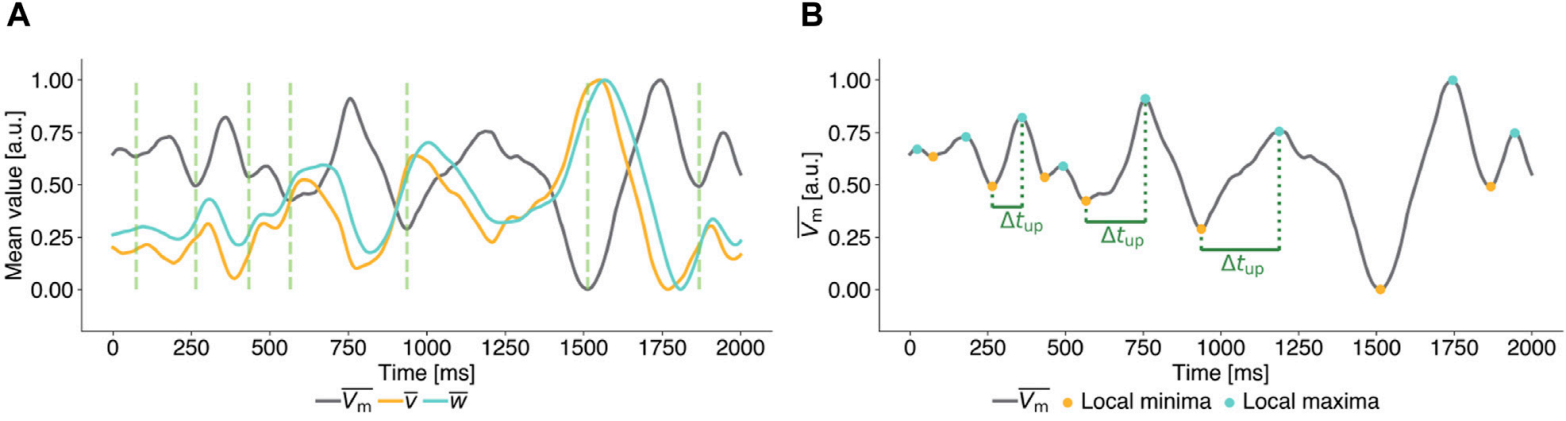
Exemplary mean transmembrane potential time series of unperturbed (**(A)**, **(B)**) chaotic states for the FK model to illustrate the parameter definitions of Local Minima Pacing (LMP). All time series are scaled to [0,1] for ease of interpretation. **(A)** Time series of the mean transmembrane potential (grey) and mean values of the gating variables (yellow and cyan). Local minima in the mean value of the transmembrane potential $\bar{V_{\text{m}}}$ (vertical dashed green lines) are followed by local maxima in the mean value of the gating variables $\bar{v}$ and $\bar{w}$ . **(B)** The upward deflection time Δ*t*_up_ (green) past local minima (yellow dots) in $\bar{V_{\text{m}}}$ is used to define the timings of the applied pulses in LMP.

We intend to contribute to the advancement of feedback-controlled protocols by utilizing the mean value of the transmembrane potential *V*_m_

$$\bar{V_{\text{m}}}=\frac{1}{N^{2}}\overset{N}{\underset{i=1}{\sum }}\overset{N}{\underset{j=1}{\sum }}V_{\text{m}}^{\text{i,j}}$$ (4)

as the observable to calculate pulse timings. Figure 2A illustrates, that local minima of $\bar{V_{\text{m}}}$ are followed by local maxima of $\bar{v}$ and $\bar{w}$ for the FK model. Intuitively, these local maxima of the mean values of the gating variables indicate times at which the system exhibits a high degree of excitability. Pulses applied to the system at these points in time are therefore more likely to excite a larger fraction of the domain than pulses at other times. The same phenomenon can be observed in the AP, BOCF, and MSA model (see Supplementary Material). This observation motivates our choice to use local minima of $\bar{V_{\text{m}}}$ as marker points to define the timings, at which pulses should be applied. We define the upward deflection time Δ*t*_up_ as the time between a local minimum and the consecutive local maximum in $\bar{V_{\text{m}}}$ (compare Figure 2B). The median $\Delta t_{\text{median}}=\tilde{\Delta t_{\text{up}}}$ of all upward deflection times over all initial conditions of a specific model of cardiac dynamics is calculated based on the last 2 seconds of $\bar{V_{\text{m}}}$ prior to the first pulse. Therefore, we determined the median upward deflection time for each model of cardiac dynamics, separately.

In Local Minima Pacing (LMP), a pulse is applied shortly after a local minimum of $\bar{V_{\text{m}}}$ is detected. Subsequently, the system evolves autonomously, the feedback of the system to the given pulse is measured and a consecutive pulse is applied after the next minimum is detected. The key parameter of LMP is the temporal distance between a local minimum and the application of a pulse, which we define with respect to the median upward deflection time. A pulse is applied if $\partial_{t}\bar{V_{\text{m}}}(t)>0$ , *∀t* ∈ [*t*_c_ − *a* Δ*t*_median_, *t*_c_] and *t*_c_ − *t*_last,p_ > *t*_pause_, where *t*_c_ is the current time, *a* acts as a scaling factor, *t*_last,p_ is the time of the last LMP pulse, and *t*_pause_ prevents consecutive pulses during an upward deflection of $\bar{V_{\text{m}}}$ initiated by a LMP pulse. Viable parameter selections for *a* are analysed in Section 3.2. LMP stops if a maximum number of pulses *max*_pulses_ is reached. By definition, however, it also stops if $\underset{\text{i,j}}{\max}V_{\text{m}}^{\text{i,j}}<\epsilon$ and therefore if all spiral wave patterns are terminated successfully. A description of the LMP approach is also given in Algorithm 1.

Algorithm 1 Local Minima Pacing - Pseudocode.

**Input:** $\bar{V_{\text{m}}}$ Mean value of the transmembrane potential
**Input:** Δ*t*_median_ Median upward deflection time.
**Input:** *a* Scaling factor.
**Input:** *t*_pause_ Pause between two consecutive pulses.
**Input:** *max*_pulses_ maximum number of pulses to apply.
  *num*_*p*_ ← 0, *t*_last,p_ ← 0
  **while** not successful AND *num*_*p*_ < *max*_pulses_ **do**
   **if** *t*_c_ − *t*_last,p_ > *t*_pause_ **then**
     **if** $\partial_{t}\bar{V_{\text{m}}}(t)\geq 0$ , *∀t* ∈ [*t*_c_ − *a* Δ*t*_median_, *t*_c_] **then**
      Apply pulse
      *t*_last,p_ ← *t*_c_
      *num*_*p*_ ← *num*_*p*_ + 1
    **end if**
   **end if**
  *t*_c_ ← *t*_c_ + Δ*t*
 **end while**

## 3 Results

In this section we present the outcomes derived with the Local Minima Pacing protocol, an investigation of optimal parameterisations of LMP for different models of cardiac dynamics, a comparison of the derived pulse timings with existing protocols, and a sensitivity analysis of the coverage rate *A*_cov_.

### 3.1 Applying pulses past local minima

**FIGURE 3:**
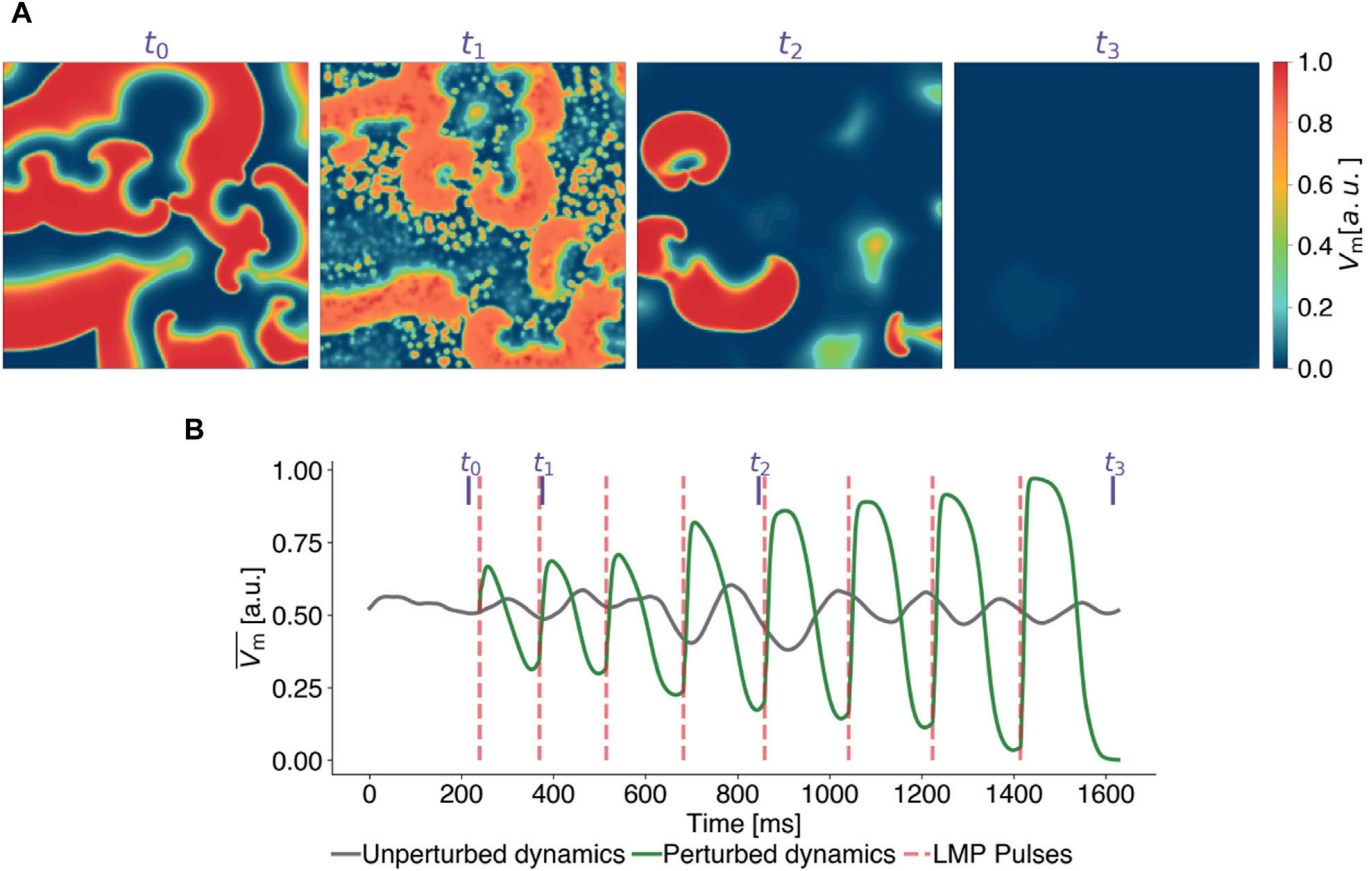
Exemplary termination attempt with Local Minima Pacing (LMP) for the Fenton-Karma model. **(A)** Snapshots of the transmembrane potential *V*_m_ at different points in time, corresponding to the purple vertical lines in subplot **(B)** where time series of the mean value of the transmembrane potential of the unperturbed (grey) and perturbed (green) dynamics are shown. LMP pulses (red lines) are applied shortly (0.2 Δ*t*_median_) after local minima are detected in $\bar{V_{\text{m}}}$ .

In this section, we begin to systematically study LMP, using a specific parameterization of it to understand how LMP pulses influence the dynamics of a system that exhibits spiral wave patterns. Figure 3 demonstrates the timing and impact of LMP pulses on the membrane potential (see Figure 3A) and the mean value of the membrane potential (Figure 3B) of the same state for the FK model in comparison to a temporal evolution without perturbations. For the analysis of the LMP algorithm presented in this subsection, we set the scaling factor for the pulse timings to *a* = 0.2, implying that pulses were applied at 20% of the median upward deflection time Δ*t*_median_ after a local minima in $\bar{V_{\text{m}}}$ was detected.

**FIGURE 4:**
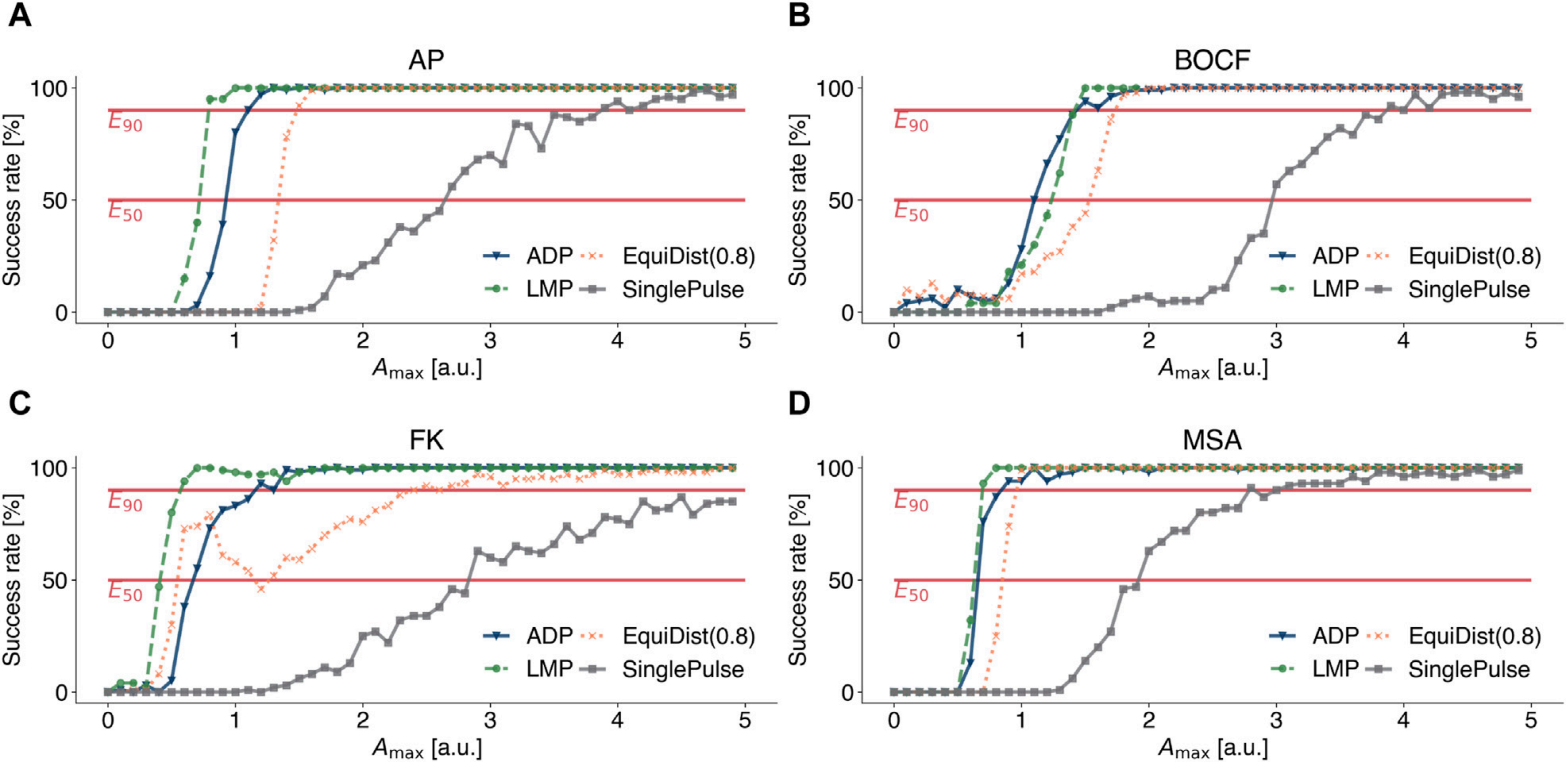
Dose-response curves for conventional defibrillation (grey squares), Equidistant Pacing (orange crosses), Adaptive Deceleration Pacing (blue triangles), and Local Minima Pacing (green circles) for **(A)** the Aliev-Panfilov (AP) model, **(B)** the Bueno-Orovio-Cherry-Fenton (BOCF) model, **(C)** the Fenton-Karma (FK) model, and **(D)** the Mitchell-Schaeffer model with the adaptions made by Alvarez et al. (MSA). The system was observed for 5 seconds to determine the pacing frequencies for Equidistant and Adaptive Deceleration Pacing. For Local Minima Pacing, the system was observed for only 2 seconds and the temporal distance between a local minimum and the application of a pulse was set to 0.2 Δ*t*_median_.

To compare the efficiency of LMP with other previously published pacing protocols, namely, conventional defibrillation (SinglePulse), Equidistant Pacing (EquiDist), and Adaptive Deceleration Pacing (ADP), we calculated dose-response curves, shown in Figure 4, for the AP model (see Figure 4A), the BOCF model (see Figure 4B), the FK model (see Figure 4C), and the MSA model (see Figure 4D), respectively. This way, a relation between the pulse amplitude *A*_max_ and the success rate to terminate spiral wave dynamics can be determined. The dominant frequency and the frequency spectrum used in EquiDist and ADP were determined on 5 s time series of the underlying dynamics, as described by Lilienkamp et al., 2022b. The pacing frequency of *EquiDist*(*c*) was set to *c f*_dom_, with *c* > 0 as a scaling factor. The local minimum of the *EquiDist* (0.8) dose response curve of the FK model at *A*_max_ ≈ 1.2 [*a*.*u*.] (see Figure 4C), was previously discussed by Lilienkamp et al., 2022a and we can confirm the reasoning of Buran et al., 2017 that later pulses may reinitiate fibrillation after successful termination.

### 3.2 Systematic investigation of pulse timings past local minima

We investigated how the scaling parameter *a* and therefore the temporal distance between a local minimum and a LMP pulse affects the overall performance of the LMP protocol. The tests we carried out cover a range of 10%–100% of the median upward deflection time Δ*t*_median_ as the timing of the LMP pulses. Motivated by decelerating pulse frequencies in the ADP protocol, we allow for increasing or decreasing scaling factors in LMP and incorporate an adjustment of the scaling factor *a*. After a LMP pulse is applied, the scaling factor is adjusted using *a* = *clamp* (*a b*, Δ*t*_start_/Δ*t*_media_, Δ*t*_end_/Δ*t*_median_), with *b* > 0 as an adjustment factor for the pulse timings during LMP. Here, Δ*t*_start_ describes the temporal difference between the first and second LMP pulse and Δ*t*_end_ between the last two pulses, respectively. This update rule is independent of the maximum number of pulses *max*_pulses_, permits an adaptive temporal distance between a local minimum in $\bar{V_{\text{m}}}$ and a LMP pulse but retains the central concept of LMP: an excitation of an increasing fraction of the domain is more likely right after local minima in $\bar{V_{\text{m}}}$ .

**FIGURE 5:**
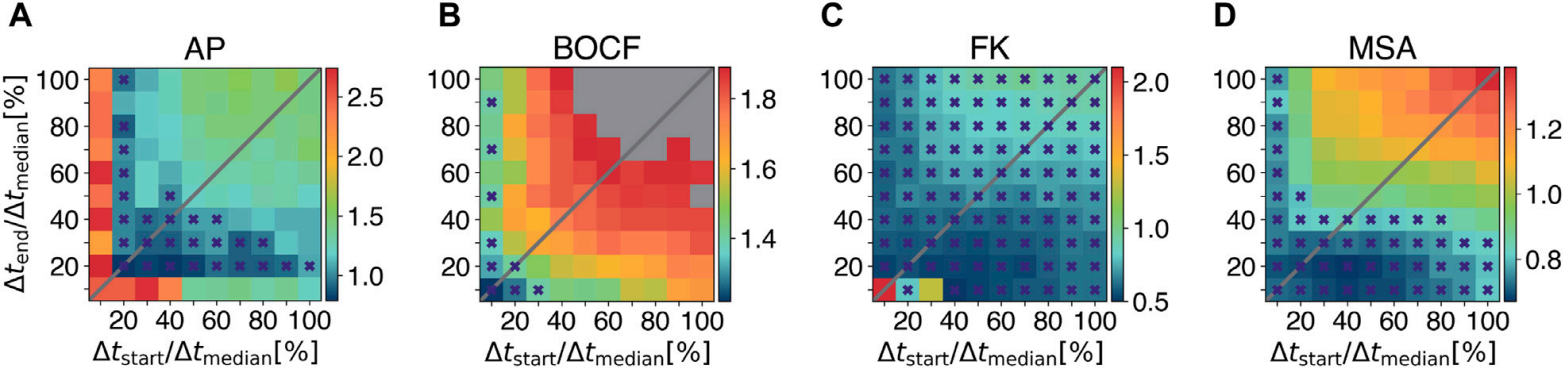
Systematic investigation of pulse timings for the Local Minima Pacing. Results for different pulse timings with respect to the required amplitude *A*_max_ to obtain a 90% success rate (colorbars) are shown for **(A)** the Aliev-Panfilov (AP) model, **(B)** the Bueno-Orovio-Cherry-Fenton (BOCF) model, **(C)** the Fenton-Karma (FK) model, and **(D)** the Mitchell-Schaeffer model with the adaptions made by Alvarez et al. (MSA), respectively. Grey squares indicate that *E*_90_ could not be achived with the given values of Δ*t*_start_ and Δ*t*_end_. Purple crosses mark combinations of Δ*t*_start_ and Δ*t*_end_ for which the required E_90_-energy of LMP is lower compared to all other protocols under investigation.

Figures 5A–D show a quantitative comparison of the pulse timings with respect to the required pulse amplitudes *A*_max_ to obtain a 90% success rate. To account for the non-linearity of the update rule, we set *b* = 0.8 if Δ*t*_start_ > Δ*t*_end_ and *b* = 1.25 if Δ*t*_start_ < Δ*t*_end_, where Δ*t*_start_ denotes the temporal difference between the first local minimum and the first LMP pulse in *ms*, Δ*t*_end_ for the last minimum and LMP pulse, respectively. We implemented ADP and EquiDist to perform pulse sequences of ten pulses. Therefore, the maximum number of LMP pulses was set to *max*_pulses_ = 10. Figures 5A–D show, that for each model multiple combinations of Δ*t*_start_ and Δ*t*_end_ exist, for which the required E90-energy of LMP is lower compared to all other protocols (marked with purple crosses). However, the key point of these plots is, that LMP with the combination of Δ*t*_start_ = Δ*t*_end_ = 0.2 Δ*t*_median_ offers an energy reduction for all models of cardiac dynamics described in Section 2.1.2. To achieve a 90% success rate, we observed a reduction in the required amplitude *A*_max_ of 28% for the AP model, 0.7% for the BOCF model, 56% for the FK model, and 18% for the MSA model compared to the ADP protocol in our numerical simulations.

### 3.3 Comparison of LMP pulse timings with other protocols

**FIGURE 6:**
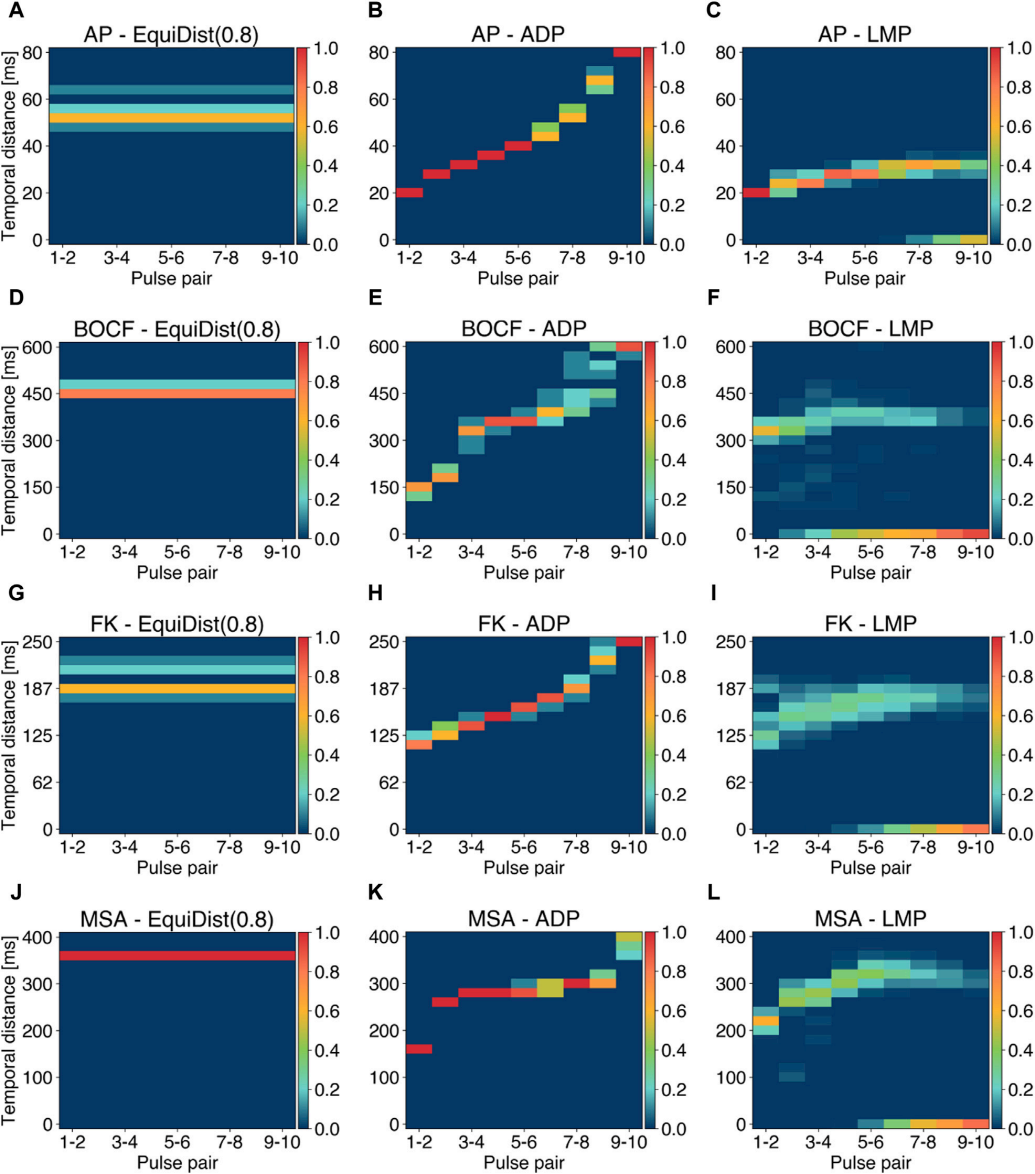
Analysis of pulse periods (temporal distance between two consecutive pulses) for the EquiDist, ADP, and LMP pacing protocols. Each column of a single plot denotes the pulse pair within a single pacing protocol, while the relative occurence of a given temporal distance (*y*-axis) over all initial conditions and all termination attempts is color coded. Plots **(A)**, **(D)**, **(G)**, and **(J)** reflect the distribution of 0.8 *f*_dom_. Plots **(B)**, **(E)**, **(H)**, and **(K)** show the distribution of periods generated using the ADP approach. Plots **(C)**, **(F)**, **(I)**, and **(L)** visualize the *a posteriori* calculated LMP periods derived with the E90-amplitude. Occurrences of 0 ms at given pulse pairs indicate successful termination without applying further pulses.

Previously published pacing protocols like EquiDist and ADP are mainly parameterized through the pulse timings of a pulse sequence (the temporal distance between consecutive pulses as depicted in Figure 3B). The proposed LMP approach, however, is parameterized by the temporal difference between a local minimum in $\bar{V_{\text{m}}}$ and a LMP pulse. To deepen our insight into the LMP approach we fixed the scaling factor to *a* = 0.2, performed an *a posteriori* analysis of the temporal distances (periods) between LMP pulses at the respective E_90_-amplitude and compared them with pulse timings from the EquiDist and ADP protocols. The relative occurrences for a given temporal distance are shown in Figure 6, where models of cardiac dynamics are displayed in separate rows and different pacing protocols in columns, respectively. The dominant period and the underlying frequency spectra of the ten initial conditions we simulated for each model of cardiac dynamics vary slightly. Therefore, the pacing periods derived by EquiDist and ADP show bundled patterns (see first and second column of Figure 6). LMP was parameterized on averages of Δ*t*_median_ of each model, and not specified on observations of each initial condition prior to pacing like EquiDist and ADP. On average, the resulting LMP periods show a decelerating pattern (compare second and third column of Figure 6), because the action potential durations increase with a growing number of LMP pulses (compare Figure 3B) but saturate at a level, that represents the action potential duration of a plane wave. It should be noted that LMP is not limited to strictly decelerating sequences of pulses. We also observe single pulse sequences that show monotonously increasing pulse periods and sharply decreasing periods in the last two pulses. Furthermore, LMP periods cover a broader range in each pulse pair compared to ADP. This observation highlights the adaptive nature of LMP, which incorporates the feedback of the underlying dynamics during pacing.

Distinct gaps in the period distribution in single pulse pairs, as shown in Figure 6I between 170–180 ms, indicate occurrences of saddle points instead of local minima of $\bar{V_{\text{m}}}$ in a subset of termination attempts. Filled period bins at 0 ms for given pulse pairs indicate successful termination without applying further pulses. For the AP (see Figure 6C), the BOCF (see Figure 6F), the FK (see Figure 6I), and the MSA (see Figure 6L) model, 50% of all successful termination attempts only required nine, five, eight, and seven pulses, respectively. Therefore, LMP not only permits the usage of lower amplitudes but also potentially reduces the number of pulses required for successful termination of the underlying dynamics. This is not the case for previously discussed termination strategies like sequences of equidistant pulses or the ADP protocol. Buran et al., 2017 describe a reinitialisation of spiral waves by later pulses in EquiDist after successful termination, which we can confirm for EquiDist and ADP. Due to its design, LMP is not susceptible to behaviour.

The feedback-controlled pacing protocol proposed by (Buran, 2023) determines pulse timings based on minima in the length of the refractory boundary *L*_RB_. These minima, however, do not always coincide with the minima in $\bar{V_{\text{m}}}$ . This observation is in line with the findings of (Buran, 2023): The correlation between the length of the refractory boundary *L*_RB_ and the excitable fraction of the domain either do not exist at all or only in a weak manner, for systems exhibiting spatiotemporal chaos and unstable spirals.

### 3.4 Sensivity analysis of the modelled coverage area

**FIGURE 7:**
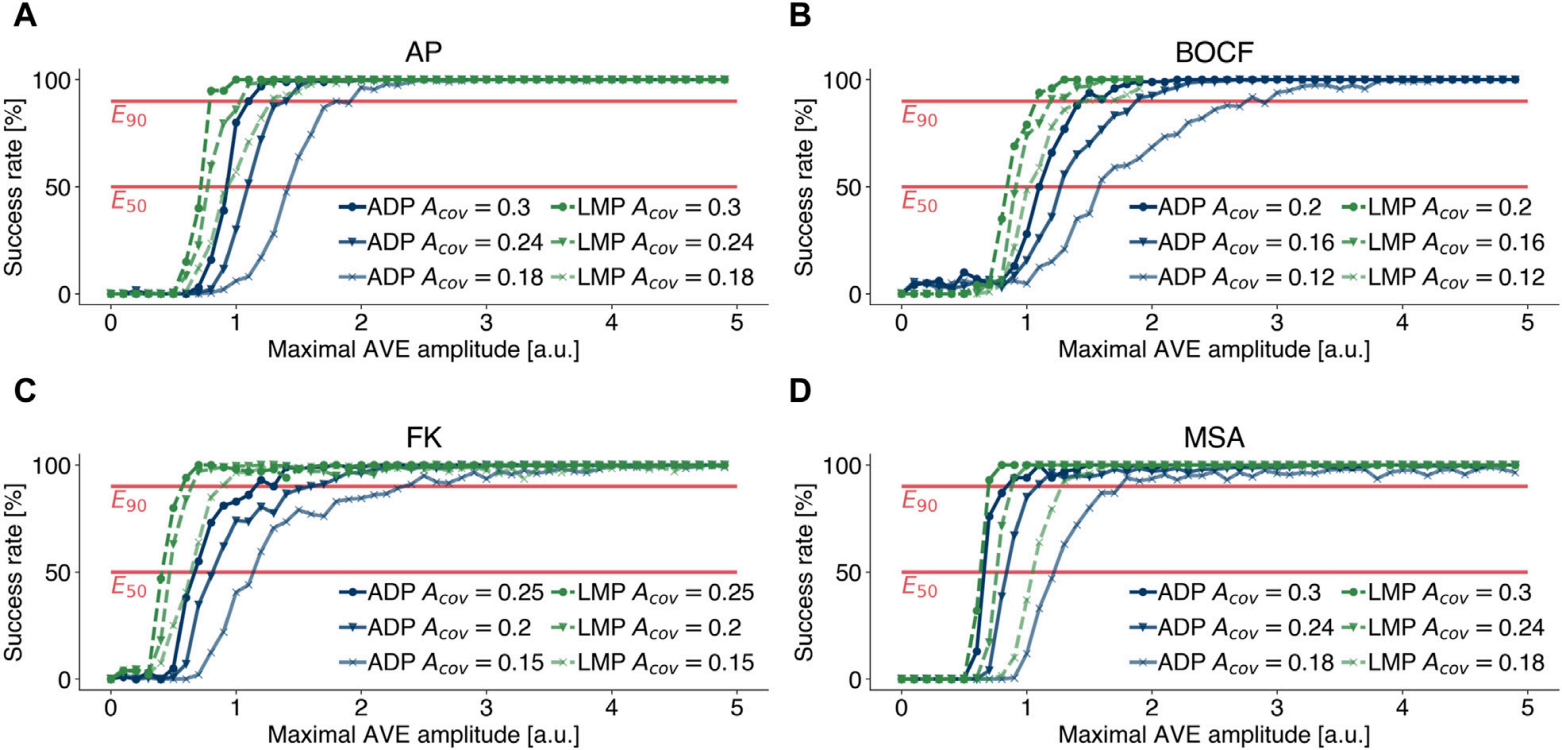
Effect of lower (marker: circle, triangle, cross) coverage rates *A*_cov_ on dose response curves of LMP (green) and ADP (blue) for **(A)** the Aliev-Panfilov (AP) model, **(B)** the Bueno-Orovio-Cherry-Fenton (BOCF) model, **(C)** the Fenton-Karma (FK) model, and **(D)** the Mitchell-Schaeffer model with the adaptions made by Alvarez et al.(MSA), respectively. Lower coverage areas require higher amplitudes *A*_max_ to achieve similar success rates.

As described in Section 2.1.4, the coverage area *A*_cov_ describes the relative fraction of the simulation domain that is covered by AVEs and was modelled very differently in previous publications (Gray, 2002; Buran et al., 2017; Lilienkamp et al., 2022b). Like other modelling parameters, the coverage area *A*_cov_ can only be approximated. There is no guarantee that this approximation applies to every individual, which is why it is important to perform a sensitivity analysis in order to be able to assess the statements based on the underlying experiments (Pathmanathan et al., 2019; Salvador et al., 2023). Therefore, we extended our investigations, presented in the previous subsections, and performed termination attempts at 80% and 60% of the coverage rates stated in Table 1 and examined how sensitive dose response curves of different pacing protocols and model of cardiac dynamics depend on different values of *A*_cov_. Figure 7 illustrates the effect of lower coverage areas *A*_cov_ and therefore lower numbers of AVEs *N*_ave_, as we kept the size of single AVEs constant as stated in Table 1. Lower coverage areas require higher amplitudes *A*_max_ to achieve similar success rates. Figures 7A–D emphasise that the aforementioned results change quantitatively, but not qualitatively, with lower coverage rates *A*_cov_. Figures that include dose response curves for EquiDist and ADP are given in the Supplementary Material.

## 4 Discussion

In this section, we outline the limitations and constraints of our study, highlight areas where further research is needed, and summarise our findings and their implications with respect to the heart as an essential part of the cardiovascular network.

### 4.1 Limitations

The numerical experiments presented in this work were performed on a two dimensional grid, on simplified models of cardiac dynamics, and without the incorporation of the muscle fibre orientation. Although the proposed approach is not yet designed to target a specific type of arrhythmia, by choosing a two-dimensional grid, our conclusions can be more directly applied to the thin, quasi-two-dimensional atrial wall (Luther et al., 2011). In particular, with respect to the approximation of the mean value of the transmembrane potential, further studies are needed to evaluate the performance of the proposed approach and the approximation of this observable on the surface of a three-dimensional grid representing the thick ventricular wall.

Furthermore, we neglected the coupling between cardiac mechanics and electrical dynamics (Ambrosi et al., 2011) in the myocardium, which influences the stability of spiral/scroll waves (Hu et al., 2013), and the shape of the action potential, especially during pacing (Hazim et al., 2021). We refer to successful defibrillation as the absence of chaotic patterns in the transmembrane potential, however, mechanical synchrony and function of the heart do not necessarily require electrical synchrony (Leclercq et al., 2002). In a further study, the proposed LMP approach should be tested in this regard.

The effect of unpinning stable spiral waves from tissue heterogeneities as a mechanism to terminate cardiac arrhythmias (Ripplinger et al. (2006)), has been studied extensively in numerical (Pumir and Krinsky 1999) and experimental (Gray and Chattipakorn, 2005; Ripplinger et al., 2006; Shajahan et al., 2016) setups.

We assume homogeneous and isotropic diffusion without conduction heterogeneities of the given scale. We therefore assume absence of scar tissue and neglect the influence of the fibre orientation (Hooks et al. 2007) in the myocardium. The interplay between LMP with the effect of unpinning, scar tissue and the influence of fibre orientation needs to be further investigated. In a future study addressing these limitations, it will be necessary to base the simulations on experimental data from specific mammals or humans.

Heterogeneities in the electrical conductivity of the myocardium are responsible for the depolarization of the transmembrane potential induced by an external electric field (Efimov et al., 2000; Pumir et al., 2007; Luther et al., 2011; Bittihn et al., 2012). In this study, this underlying mechanism is modelled by the application of local current injections at predefined locations within the simulation domain (Lilienkamp et al., 2022b), referred to as artificial virtual electrodes (AVEs). By employing the concept of AVEs, we accept that in particular the first milliseconds of the interaction between an external electric field and the myocardium is simplified. In previous studies, the utilization of AVEs proved effective in replicating the amplitude reductions of applying sequences of pulses at constant pulse timings in contrast to conventional defibrillation (Lilienkamp et al., 2022a; Lilienkamp et al., 2022b), which was also shown in other numerical (Buran et al., 2017) and experimental (Fenton et al., 2009) studies. Furthermore, applying sequences of low energy pulses may lead to the presence of local minima in the dose response curves for different models of cardiac dynamics (Buran et al., 2017), which can also be reproduced using the concept of AVEs (Lilienkamp et al., 2022a; Lilienkamp et al., 2022b). The aim of this study is therefore not to predict the exact shock strengths required by different pacing protocols, but rather to make qualitative statements about possible amplitude reductions of the protocols with respect to each other. However, this concept enables a grid spacing (Δ*x*, *N*, and Δ*t*) to perform a large number of simulations and thus a statistical analysis of the presented methods on multiple models of cardiac dynamics.

### 4.2 Summary

In this study, we investigated a novel approach as a promising alternative for low-energy defibrillation of cardiac arrhythmias like ventricular fibrillation. Multiple approaches, like equidistant pacing (Fenton et al., 2009; Luther et al., 2011) and adaptive deceleration pacing (Lilienkamp et al., 2022b), have been proposed to reduce the energy required for successful defibrillation. However, an energy reduction that enables defibrillation without side effects remains an open challenge.

In this study, we showed that pulses shortly after local minima in the mean value of the transmembrane potential can provide a significant energy reduction compared to recently published pacing protocols. Although the dynamics of the underlying system during pacing is incorporated in this approach, a single parameterization (scaling factor *a* = 0.2) of the proposed algorithm could be identified that provides a reduction of the energy for all investigated models of cardiac dynamics. This observation suggests that this parameterization may also result in energy reductions in future *ex vivo* experiments. Moreover, we provide a sensitivity analysis to demonstrate stability of the drawn conclusions with respect to a modelling parameter that was not selected uniformly in earlier publications. Compared to EquiDist and ADP, LMP potentially reduces the number of pulses and therefore the total electrical energy applied required to successfully terminate the underlying dynamics. Furthermore, as LMP incorporates the feedback of the underlying dynamics, a broader frequency band within each pulse pair is covered. This observation highlights the adaptive nature of this approach with respect to the underlying dynamics during pacing.

LMP solely relies on the measurement of the mean value of the transmembrane potential, an observable that can be measured very easily in numerical simulations, compared to the length of the refractory boundary (used in the feedback-controlled pacing algorithm proposed by Buran, 2023). As the measurement or approximation of both observables remains an open challenge in *ex vivo* experiments, we suggest testing the feasibility and reliability of these approximations in future experiments. In order to transfer this approach to more realistic numerical or even living-heart experiments, the mean value of the transmembrane potential needs to be computed, which remains an open challenge. We propose to reconstruct and approximate this observable by reconstructing the electrical activity of the heart from densely sampled body-surface potentials (Bear et al., 2018; Xie and Yao, 2022) in future studies. Of particular interest will be the noise induced by these approximation techniques and its influence on the minima and maxima of the mean transmembrane potential. The application of controlled electrical stimuli during LMP may further complicate the live measurement of the mean value of the transmembrane potential, e.g., by interactions between the external electric field and the ECG electrodes.

Characteristic features of atrial (AF) and ventricular fibrillation (VF) cover a broad range, depending on the biological species, medical condition and other factors. The mean dominant frequency of the underlying dynamics range from *f*_dom_ = 6.2 ± 0.2 Hz in the absence of cardiogenic shock and *f*_dom_ = 4.0 ± 0.2 Hz in the presence of cardiogenic shock for human VF (Stewart et al., 1992), *f*_dom_ = 5.7 ± 1.2 Hz for human AF (Bollmann et al., 1998), *f*_dom_ = 9.4 ± 2.6 Hz for AF in isolated sheep hearts (Skanes et al., 1998) to *f*_dom_ = 13.2 ± 3.4 Hz in isolated rabbit hearts (Chen et al., 2000). With regard to the mean dominant frequencies, this should provide a motivation for our choice of parameterizations of the models investigated, as these cover a range between *f*_dom_ = 2.65 Hz (BOCF) and *f*_dom_ = 22.46 Hz (AP). Furthermore, the mean number of phase singularities in our simulations range from *N*_PS_ = 10.74 (FK) to *N*_PS_ = 22.11 (AP). Although *N*_PS_ = 22.11 phase singularities clearly only applies to outliers, this range corresponds to recent numerical and experimental studies on pig and human hearts, respectively (Nash et al., 2006; Ten Tusscher et al., 2007). 

Therefore our findings are not tailored to a specific model of cardiac dynamics, a parameterization of it, a single biological species, or one specific type of cardiac arrythmia but rather show a basic property inherent in all of these models, that can be utilized for defibrillation. Therefore, the exact energy reduction achievable with this approach, particularly taking into account the species, medical condition and type of cardiac arrhythmia, is therefore to be determined in future studies. The results presented, however, strongly indicate that LMP is a viable option to significantly lower the negative side effects associated with conventional defibrillation techniques.

## Data Availability

The raw data supporting the conclusion of this article will be made available by the authors, without undue reservation.

## Abbreviations

ADP, adaptive deceleration pacing; AP, Aliev-Panfilov model; AVE, artificial virtual electrode; BOCF, Bueno-Orovio-Cherry-Fenton model; EquiDist, equidistant pacing; FK, Fenton-Karma model; LMP, local minima pacing; MSA, Mitchell-Schaeffer model with adaptions by Alvarez; AF, atrial fibrillation; VF, ventricular fibrillation.
