# Supplementary material for: Chaos control in cardiac dynamics: terminating chaotic states with local minima pacing
Source: Front Netw Physiol. 2024 Jul 3;4:1401661. doi: 10.3389/fnetp.2024.1401661 (PMC11252590; doi:10.3389/fnetp.2024.1401661)
Supplement: Supplementary file 1 [file DataSheet1.ZIP › Frontiers_LMP_Supplements.pdf]

Supplementary Material

1 CARDIAC CELL MODELS

To ensure the comparability of the results presented in this manuscript, we applied the same parameter sets as Lilienkamp et al. (Lilienkamp et al. (2022)) for the investigated models.

1.1 Aliev-Panfilov

The Aliev-Panfilov model (Aliev and Panfilov (1996)) is a two-variable model. We used the parametrization as given in Table S1.

Table S1. Parametrization of Aliev-Panfilov model.

Parameter	Value	Parameter	Value
a	0.06	μ_1	0.2
k	10.0	μ_2	0.3
ϵ_0	0.001		

1.2 Bueno-Orovio-Cherry-Fenton

The Bueno-Orovio-Cherry-Fenton model (Bueno-Orovio et al. (2008)) is a four-variable model. We used the parametrization denoted as PB in the original paper.

1.3 Fenton-Karma

The Fenton-Karma model (Fenton and Karma (1998)) is a three-variable model. We used the parametrization as given in Table S2.

Table S2. Parametrization of Fenton-Karma model.

Parameter	Value	Parameter	Value	Parameter	Value
τ_{v+}	$13.03ms$	τ_0	$12.5ms$	τ_{v1}^-	$1250ms$
τ_r	$33.25ms$	τ_{v2}^-	$19.6ms$	τ_{si}	$29ms$
τ_w^+	$800ms$	u_c	$0.13a.u.$	τ_w^-	$40ms$
C_m	$1.0\mu F/cm^2$	τ_d	$0.45ms$	u_v	$0.04a.u.$
u_c^{si}	$0.85a.u.$				

1.4 Mitchell-Schaeffer

The Mitchell-Schaeffer model (Álvarez et al. (2012)) is a two-variable model. We used the parametrization and adoptions made by Alvarez as stated in the original paper.

2 INITIALIZATION OF SPIRAL WAVE CHAOS

To initialize chaotic states, we initialized a single spiral wave at $t = 0.0ms$, by setting the membrane potential $V_\mathrm{m} = 1.0$ in the upper fraction (AP: half, BOCF: three quarters, FK: quarter, MSA: half) of the simulation domain and setting the gating variables to $h = 1 - h_0$ (where h_0 describes the resting state value

Table S3. Parameters used to initialise initial conditions.

Model	$t_s[ms]$	$size_s[mm^2]$	num_s	$\delta_{t,i}[ms]$
AP	500	10	20	5500
BOCF	750	40	20	5250
FK	250	20	10	5750
MSA	1000	16	10	6000

of the gating variable) in the left/right half respectively. At time $t = t_s$ we applied num_s smaller spirals as described above at random positions of the domain (here, the fractions refer to the given mask sizes $size_s$ in Table S3). Video material showing exemplary initial conditions created this way can also be found in the supplements. To ensure independence of the initial conditions generated this way, we evolved each state for another time period $\delta_{t,i}$. Each initial condition was tested for another 5 seconds, to exclude self-terminating states. The timings t_s, mask sizes $size_s$, and number num_s of these spirals are given in Table S3.

3 LOCAL MINIMA IN PSEUDO ELECTROCARDIOGRAMS OF DIFFERENT CARDIAC CELL MODELS

Figure S1 illustrates the $pECG$ values for each variable and all models under investigation.

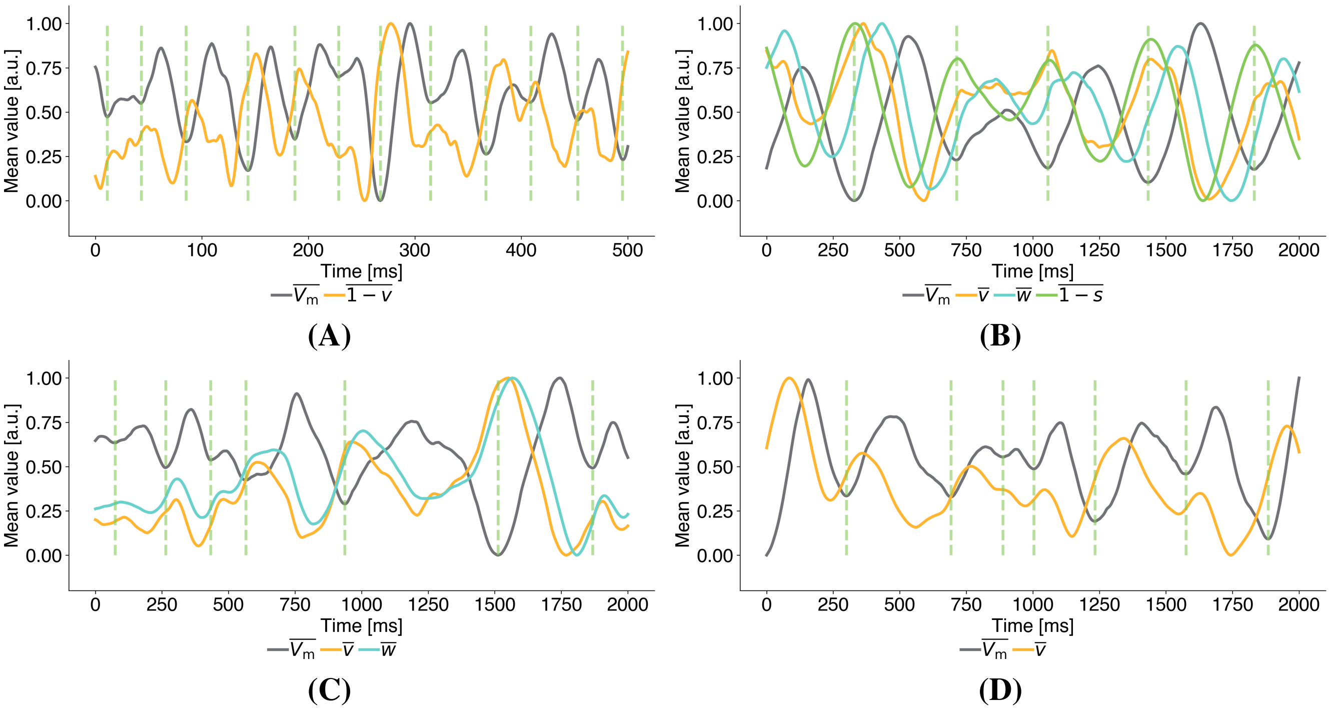

Figure S1. Exemplary pseudo ECG time series of chaotic states **(A)** AP model, **(B)** BOCF model, **(C)** FK model, and **(D)** MSA model.

4 SENSIVITY ANALYSIS OF THE MODELLED COVERAGE AREA

Figure S2 represents the dose response curves for all protocols and cardiac cell models under investigation at different coverage areas A_{cov}.

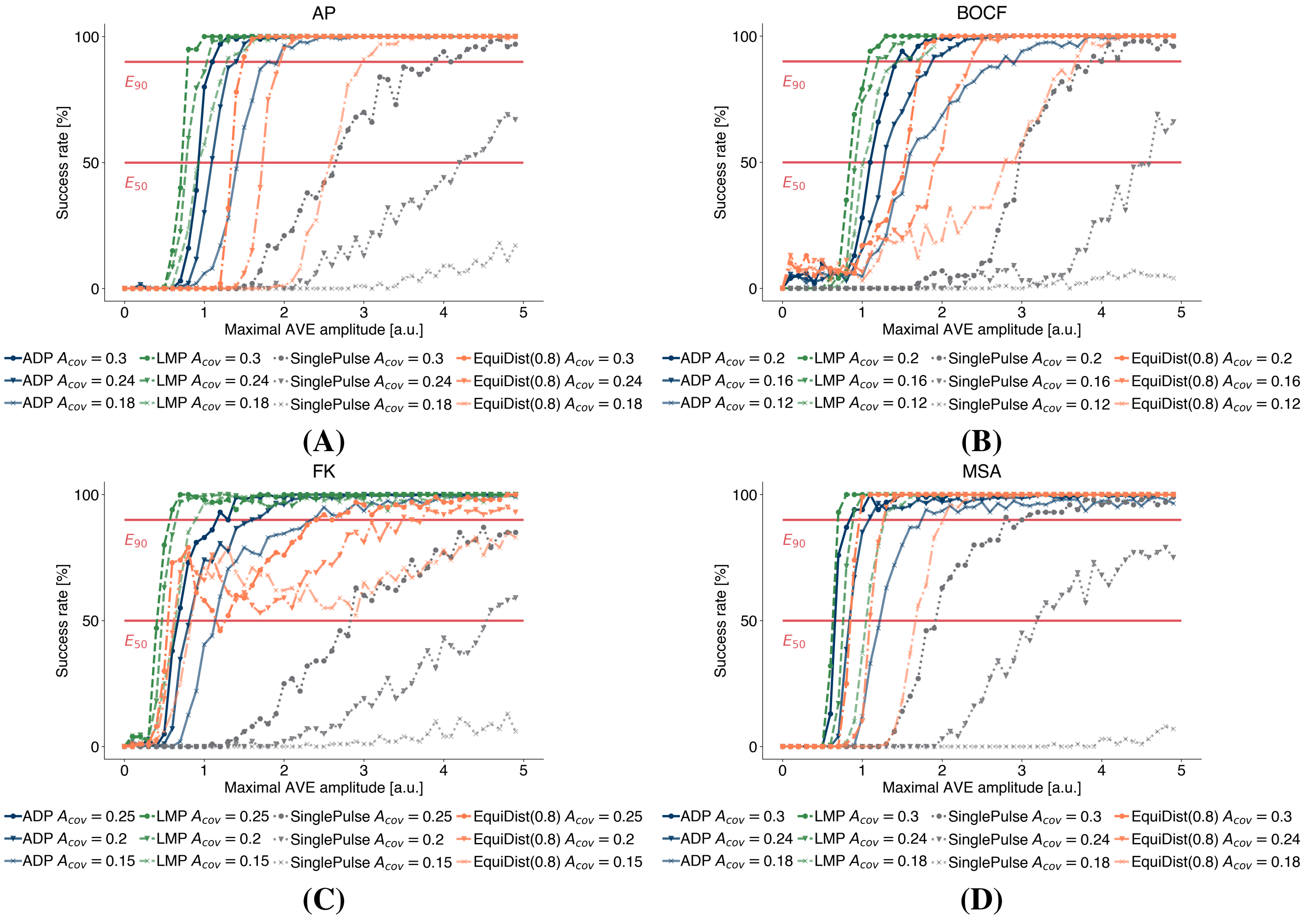

Figure S2. Effect of lower (marker: circle, triangle, cross) coverage rates A_{cov} on dose response curves of SinglePulse (grey), EquiDist(0.8) (orange), LMP (green), and ADP (blue) for different cardiac cell models. Lower coverage areas require higher locally injected currents A_{max} to achieve similar success rates.

REFERENCES

Aliev, R. R. and Panfilov, A. V. (1996). A simple two-variable model of cardiac excitation. *Chaos Solitons Fractals* 7, 293–301

Álvarez, D., Alonso-Atienza, F., Rojo-Álvarez, J. L., García-Alberola, A., and Moscoso, M. (2012). Shape reconstruction of cardiac ischemia from non-contact intracardiac recordings: A model study. *Math. Comput. Model.* 55, 1770–1781

Bueno-Orovio, A., Cherry, E. M., and Fenton, F. H. (2008). Minimal model for human ventricular action potentials in tissue. *J. Theor. Biol.* 253, 544–560

Fenton, F. and Karma, A. (1998). Vortex dynamics in three-dimensional continuous myocardium with fiber rotation: Filament instability and fibrillation. *Chaos* 8, 20–47

Lilienkamp, T., Parlitz, U., and Luther, S. (2022). Taming cardiac arrhythmias: Terminating spiral wave chaos by adaptive deceleration pacing. *Chaos: An Interdisciplinary Journal of Nonlinear Science* 32
